# Narrative therapy and resilience training improve recovery and survival after intersphincteric resection for low rectal cancer: a randomized trial

**DOI:** 10.1093/oncolo/oyaf308

**Published:** 2025-11-04

**Authors:** Gang Wang, Shengjie Pan

**Affiliations:** Department of General Surgery, The First Affiliated Hospital of Soochow University, Suzhou City, Jiangsu Province 215006, China; Department of Neurology, The First Affiliated Hospital of Soochow University, Suzhou City, Jiangsu Province 215006, China

**Keywords:** low rectal cancer, intersphincteric resection, narrative therapy, psychological resilience, postoperative recovery, survival

## Abstract

**Background:**

Patients undergoing intersphincteric resection (ISR) for low rectal cancer often experience persistent bowel dysfunction, psychological distress, and compromised quality of life, especially in the context of preventive stoma creation. These challenges can negatively affect recovery, immune function, and long-term prognosis. Psychosocial interventions, such as narrative therapy and resilience training, may mitigate these effects, yet their integrated application in ISR populations remains unexplored.

**Methods:**

In this single-center randomized controlled trial, 178 patients with stage I-III low rectal cancer who underwent ISR between October 2019 and October 2021 at the First Affiliated Hospital of Soochow University were randomized to receive either standard care or a structured 6-month intervention combining narrative therapy and resilience training. Primary outcomes included psychological resilience (CD-RISC), emotional well-being (HADS), sleep quality (PSQI), and nutritional recovery (serum albumin, prealbumin, BMI). Secondary endpoints encompassed postoperative complications, systemic inflammation (CRP, IL-6, TNF-α), and 2-year disease-free survival (DFS) and overall survival (OS). All analyses followed the intention-to-treat principle.

**Results:**

The intervention group demonstrated significantly greater improvements in psychological and nutritional parameters (all *P* < .01), fewer complications (12.4% vs. 23.6%, *P* = .035), and reduced inflammatory markers on postoperative day 7. At 24 months, both DFS (89.2% vs. 75.3%, *P* = .028) and OS (93.1% vs. 81.6%, *P* = .031) were significantly higher in the intervention group. Effect sizes (Cohen’s *d*) and minimal clinically important differences (MCIDs) were assessed to support the interpretation of clinical relevance.

**Conclusion:**

An integrated psychosocial intervention significantly enhanced functional recovery and long-term oncologic outcomes following ISR. These findings underscore the value of incorporating structured psychological support into postoperative care for low rectal cancer.

Implications for Practice Patients undergoing intersphincteric resection for low rectal cancer often face severe bowel dysfunction, emotional distress, and delayed recovery. This study shows that a structured psychosocial program—combining narrative therapy and resilience training—can improve emotional well-being, reduce complications, and even enhance survival. These findings support the integration of psychological care into surgical oncology pathways. As a low-cost, scalable approach, this intervention may help address the overlooked mental health needs of colorectal cancer patients and promote truly patient-centered recovery.

## Introduction

Low rectal cancer, defined as tumors located within 6 cm of the anal verge, presents unique therapeutic challenges due to its anatomical proximity to the sphincter complex and pelvic floor. Intersphincteric resection (ISR) offers a sphincter-preserving alternative to abdominoperineal resection, aiming to maintain continence while achieving oncologic clearance. However, the functional cost of ISR can be substantial. Postoperative low anterior resection syndrome (LARS)—characterized by fecal urgency, clustering, and incontinence—occurs in a significant proportion of patients and is often exacerbated by the creation of a temporary diverting stoma. These complications markedly diminish health-related quality of life, impair social functioning, and induce persistent psychological stress.^1^^,^^2^

Emerging evidence suggests that the psychological impact of ISR may extend beyond transient distress. Postoperative anxiety, depression, and sleep disturbances are prevalent among patients undergoing sphincter-preserving surgery and have been independently associated with impaired immune function, delayed nutritional recovery, systemic inflammation, and inferior oncologic outcomes.^3^^,^^4^ Despite these findings, routine postoperative care for rectal cancer continues to prioritize surgical and oncologic parameters while neglecting psychological adaptation as a modifiable determinant of recovery.

Psychological resilience—the dynamic ability to cope effectively with adversity—has been identified as a crucial factor influencing recovery and survivorship in cancer patients. Structured resilience training programs, grounded in cognitive-behavioral principles, have demonstrated efficacy in reducing emotional distress and promoting adaptive coping mechanisms.^5^ In parallel, narrative therapy—a patient-centered intervention that encourages individuals to reframe and reconstruct their illness narratives—has shown promise in improving emotional well-being, strengthening identity coherence, and enhancing social connectedness.^6^

Although both resilience training and narrative therapy have demonstrated benefits in cancer care, their synergistic application in the context of ISR remains unexplored. Considering the complex interplay between functional, emotional, and oncologic stressors in ISR patients, a combined psychosocial approach may offer multidimensional therapeutic gains.^7^

This randomized controlled trial was designed to evaluate the impact of an integrated psychosocial intervention—comprising structured resilience training and guided narrative therapy—on postoperative recovery and long-term outcomes in patients undergoing ISR for low rectal cancer. We hypothesized that the intervention would enhance emotional adaptation, accelerate nutritional and inflammatory recovery, and ultimately improve survival outcomes.^8^

## Methods

### Study design and participants

This single-center, parallel-group, randomized controlled trial was conducted at the Department of General Surgery, First Affiliated Hospital of Soochow University between October 2019 and October 2021. The study adhered to the CONSORT 2010 guidelines and received approval from the institutional ethics committee. All participants provided written informed consent.

Eligible participants were adults aged 18-75 years with histologically confirmed stage I-III low rectal adenocarcinoma (tumor ≤6 cm from the anal verge) who were scheduled to undergo curative intersphincteric resection (ISR). The indication for ISR was confirmed by a multidisciplinary tumor board based on oncologic feasibility and sphincter preservation potential.

Inclusion criteria included:

1.  Clinical stage I-III low rectal cancer;
2.  Indicated for ISR with or without a protective stoma;
3.  Eastern Cooperative Oncology Group (ECOG) performance status 0-1;
4.  Ability to complete psychological assessments and follow-up visits.

Exclusion criteria included:

1.  Evidence of distant metastasis;
2.  Prior pelvic radiotherapy or major rectal surgery;
3.  Diagnosed psychiatric illness, cognitive impairment, or neurodegenerative disease;
4.  Enrollment in another intervention trial;
5.  Anticipated nonadherence or logistical inability to complete intervention sessions.
6.  Age >75 years, due to the higher prevalence of cognitive decline, reduced physiological reserve, and multiple comorbidities, which could confound assessment of psychosocial intervention efficacy.

### Randomization and masking

After baseline assessment, eligible participants were randomly allocated (1:1) to the intervention or control group using a computer-generated block randomization sequence (block size of 4), stratified by clinical stage and stoma status. Allocation concealment was maintained using opaque, sequentially numbered envelopes prepared by an independent research coordinator. Due to the nature of the psychosocial intervention, participants and facilitators could not be blinded. However, outcome assessors and statisticians remained blinded to group assignments throughout data collection and analysis.

### Intervention

All patients received standardized perioperative management, including enhanced recovery after surgery (ERAS) protocols, nutritional guidance, and stoma care education when applicable. The ERAS pathway comprised early mobilization beginning on postoperative day 1, multimodal analgesia to minimize opioid use, early initiation of oral feeding with gradual advancement, pelvic floor rehabilitation as a routine component of the institutional ERAS program (provided equally to both groups), and structured colostomy education sessions delivered by specialized stoma nurses, in accordance with international ERAS/ESPEN guidelines.^9^

Patients in the intervention group additionally received a structured psychosocial program initiated within two weeks postoperatively and continuing for 6 months. The intervention integrated four components:

1.  Psychological resilience training (CBT-based): Conducted as individual sessions, once weekly for 60 minutes, over the first 8 postoperative weeks. The curriculum followed the Connor–Davidson resilience model, focusing on emotional regulation, adaptive cognitive restructuring, stress inoculation, goal setting, and self-efficacy enhancement.
2.  Narrative therapy: Delivered in small groups (3-5 patients), biweekly, for 90 minutes each, between weeks 2 and 12 after surgery. Sessions used a semi-structured format, facilitating storytelling, reflective discussion, and expressive writing to help patients reconstruct illness experiences, strengthen identity integration, and foster social connectedness.
3.  Family education and support: Provided as monthly group seminars (60 minutes) during months 1, 2, 3, 6, and 12, supplemented with printed handouts. These sessions addressed emotional support strategies, nutritional guidance, and stoma adaptation.
4.  Follow-up phone counseling: Conducted every 2 months (20-30 minutes per call) during months 3-11 to reinforce coping skills, monitor psychosocial well-being, and provide continuous support.

Intervention fidelity was maintained through standardized manuals and regular supervision meetings. Session attendance and engagement levels were systematically recorded. A detailed schedule of intervention components and timepoints is presented in Supplementary Table S1.

### Outcome measures

All outcomes were assessed at baseline and postoperatively at 1, 3, 6, 12, and 24 months by trained evaluators blinded to group assignment.

Primary outcomes were:

1.  Psychological resilience: Measured using the 25-item Connor–Davidson Resilience Scale (CD-RISC)^10^;
2.  Emotional distress: Assessed via the Hospital Anxiety and Depression Scale (HADS)^11^;
3.  Sleep quality: Evaluated using the Pittsburgh Sleep Quality Index (PSQI)^12^;
4.  Nutritional status: Quantified by serum albumin (g/L), prealbumin (mg/L), and body mass index (BMI, kg/m^2^).^13^^,^^14^

Secondary outcomes included:

1.  Postoperative recovery: Length of hospital stay (days) and complication rates (Clavien–Dindo grade ≥ II)^15^;
2.  Inflammatory response: C-reactive protein (CRP), interleukin-6 (IL-6), and tumor necrosis factor-alpha (TNF-α) measured on postoperative day 7^16^;
3.  Oncologic outcomes: Two-year disease-free survival (DFS) and overall survival (OS), determined via imaging, colonoscopy, and clinical review.^17^

### Sample size calculation

Based on prior literature, we estimated a 2-year DFS of 72% in the control group and hypothesized an improvement to 86% in the intervention group. Using a two-sided α of 0.05 and 80% power, a sample size of 84 patients per group was required. Allowing for a 5% attrition rate, the final target enrollment was set at 178 patients (89 per group).

This estimation was informed by prior observational studies linking psychological resilience and stress regulation to improved immune function and survival in gastrointestinal cancers.^18^^,^^19^ Although DFS was not the primary endpoint, we deliberately based our sample size calculation on this outcome because it was the most clinically relevant indicator of long-term benefit. This conservative calculation ensured that the study also maintained adequate statistical power to detect meaningful differences in the designated primary endpoints (psychosocial and nutritional outcomes).

### Statistical analysis

Data were analyzed using SPSS version 26.0 (IBM Corp., Armonk, NY). Continuous variables were reported as mean ± standard deviation (SD) and compared using independent-samples *t*-tests or Mann–Whitney *U*-tests, as appropriate. Categorical variables were analyzed using chi-square or Fisher’s exact tests.

Effect sizes for key between-group comparisons were calculated using Cohen’s *d*, defined as the difference in group means divided by the pooled standard deviation. Interpretation followed conventional thresholds: 0.2 (small), 0.5 (medium), and 0.8 (large). Effect sizes were primarily reported for psychosocial (CD-RISC, HADS, PSQI), nutritional (albumin, prealbumin, BMI), and inflammatory (CRP, IL-6, TNF-α) outcomes at clinically relevant timepoints (eg, 3 months). Where available, minimal clinically important differences (MCIDs) were used as additional reference points to interpret clinical relevance.

Longitudinal outcomes (eg, CD-RISC, HADS, PSQI, and nutritional markers) were analyzed using linear mixed-effects models (LMMs), incorporating group (intervention vs. control), time (categorical: baseline, 1, 3, 6, 12, and 24 months), and group × time interaction as fixed effects, with a random intercept for each participant. This approach accounted for intra-individual correlation and missingness across repeated measures. For repeated between-group comparisons across multiple timepoints, multiplicity was controlled using Bonferroni correction, consistent with the reporting in Supplementary Tables S2 and S3.

Survival outcomes (2-year disease-free survival [DFS] and overall survival [OS]) were estimated using Kaplan–Meier analysis with log-rank tests, and further assessed using multivariate Cox proportional hazards models adjusted for relevant baseline covariates (eg, age, sex, TNM stage, baseline psychosocial and nutritional scores). The proportional hazards assumption was tested using Schoenfeld residuals.

All analyses followed the intention-to-treat (ITT) principle.

Missing data were addressed using multiple imputation by chained equations (MICE) under the assumption of missing at random. The imputation model included baseline covariates (age, sex, TNM stage, stoma status), primary and secondary outcome variables, and group assignment. Five imputed datasets were generated and pooled according to Rubin’s rules.

Sensitivity analyses were conducted to assess the robustness of results, including:

1.  Complete-case analysis;
2.  Models adjusted for baseline values of outcomes;
3.  Mixed-effects models without imputation (maximum likelihood estimation).

All tests were two-sided, with a significance threshold of *P* < .05.

## Results

### Patient enrollment and baseline characteristics

**Figure 1.**
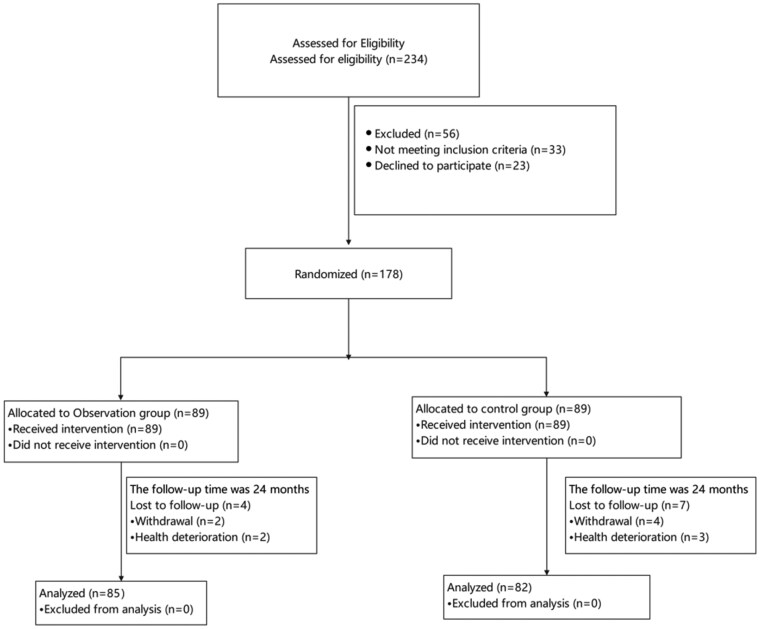
Flow chart. Notes: Flowchart depicting patient screening, randomization, group allocation, and 24-month follow-up, following CONSORT guidelines.

Between October 2019 and October 2021, a total of 234 patients with stage I-III low rectal cancer were assessed for eligibility. Of these, 178 met inclusion criteria and were randomly assigned to the intervention group (*n* = 89) or control group (*n* = 89). All patients underwent curative intersphincteric resection (ISR) and were included in the intention-to-treat (ITT) analysis. At 24 months, follow-up completion rates were 95.5% and 92.1% in the intervention and control groups, respectively (Figure 1).

Baseline demographic and clinical characteristics, including age, sex, BMI, tumor stage, surgical approach, preoperative psychosocial and nutritional scores (CD-RISC, HADS, PSQI, albumin, BMI), as well as treatment-related variables such as neoadjuvant chemoradiation (62% overall; 61.8% in the intervention group vs. 61.8% in the control group, *P* = 1.000) and protective diverting stoma creation (71.9% vs. 69.7%, *P* = .763), were comparable between the two groups (all *P* > .05; Table 1). This confirmed adequate randomization and baseline balance.

**Table 1.** Baseline demographic and clinical characteristics.

Variable	Intervention (*n* = 89)	Control (*n* = 89)	Test statistic	*P*-value
**Age (years)**	59.4 ± 8.1	59.9 ± 8.6	*F* = 0.197	.658
**Gender**			χ² = 0.123	.726
**└ Male**	57 (64.0%)	55 (61.8%)		
**└ Female**	32 (36.0%)	34 (38.2%)		
**BMI (kg/m²)**	22.8 ± 2.4	22.9 ± 2.6	*F* = 0.054	.817
**Surgical approach**			χ² = 0.091	.763
**└ Laparoscopic**	76 (85.4%)	75 (84.3%)		
**└ Open surgery**	13 (14.6%)	14 (15.7%)		
**TNM stage**			χ² = 0.178	.915
**└ Stage I**	13 (14.6%)	14 (15.7%)		
**└ Stage II**	34 (38.2%)	35 (39.3%)		
**└ Stage III**	42 (47.2%)	40 (44.9%)		
**Neoadjuvant chemoradiation**	55 (61.8%)	55 (61.8%)	χ² = 0.000	1.000
**Protective diverting stoma**	64 (71.9%)	62 (69.7%)	χ² = 0.091	.763
**Baseline PSQI**	9.0 ± 2.4	9.1 ± 2.3	*F* = 0.039	.843
**Baseline HADS**	16.0 ± 4.1	16.3 ± 4.2	*F* = 0.225	.636
**Baseline CD-RISC**	59.8 ± 9.0	60.1 ± 8.8	*F* = 0.078	.780

Notes: Baseline values are reported as mean ± standard deviation (SD) for continuous variables and number (%) for categorical variables. Between-group comparisons were conducted using independent-samples *t*-tests for continuous variables and chi-square or Fisher’s exact tests for categorical variables, as appropriate. No statistically significant differences were observed across baseline characteristics (all *P* > .05), confirming group balance post-randomization. In subsequent longitudinal analyses, ANCOVA or linear mixed-effects models were applied to adjust for relevant baseline covariates (eg, PSQI, HADS, CD-RISC scores, TNM stage, adjuvant therapy). Missing data were handled using maximum likelihood estimation under the mixed model framework. All analyses followed the intention-to-treat principle. Data on intervention fidelity and adherence are reported in the Results (*Psychosocial recovery: resilience, sleep, and emotional state* and *Nutritional recovery*).

### Psychosocial recovery: resilience, sleep, and emotional state

**Figure 2.**
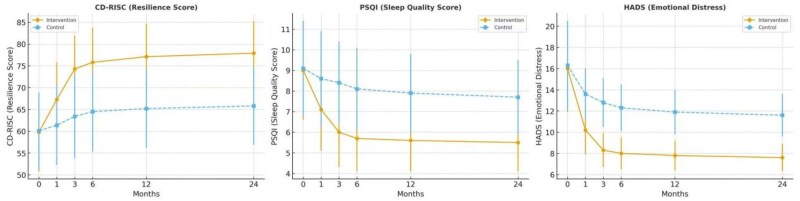
Postoperative psychological outcomes. Notes: (A) Psychological resilience, assessed using the CD-RISC, improved significantly and persistently in the intervention group compared with the control group, with the most substantial gains observed within the first 3 months postoperatively (Cohen’s *d* = 1.21 at 3 months). (B) Sleep quality, measured by PSQI scores, showed a marked improvement (ie, reduction in score) in the intervention group, whereas the control group demonstrated slower and less substantial changes (Cohen’s *d* = 1.23 at 3 months). (C) Emotional distress, evaluated via HADS, decreased significantly in the intervention group, with large between-group differences evident from 1 month and sustained through 24 months (Cohen’s *d* = 1.15 at 3 months). All data are presented as mean ± standard deviation (SD) and were analyzed using linear mixed-effects models under the intention-to-treat (ITT) principle. Error bars represent 95% confidence intervals (CIs) at each timepoint. Intervention fidelity and adherence were systematically monitored, and completion rates are reported in the Results (*Psychosocial recovery: resilience, sleep, and emotional state* and *Nutritional recovery*). Observed improvements in CD-RISC and HADS exceeded the minimal clinically important differences (MCIDs), underscoring the clinical relevance of the intervention. See Supplementary Table S2 for full timepoint data.

The intervention group exhibited significantly greater and sustained improvements in all psychosocial domains across the 24-month follow-up period (Figure 2A-C; Supplementary Table S2).

 Psychological resilience (CD-RISC): At 3 months, mean scores increased from 59.8 ± 9.0 to 74.3 ± 7.6 in the intervention group, compared with 60.1 ± 8.8 to 63.4 ± 9.5 in controls (mean difference: 10.9, 95% CI: 9.2-12.6, *P* < .001, Cohen’s *d* = 1.21). This difference also exceeded the minimal clinically important difference (MCID) of 5 points, indicating a large and clinically meaningful improvement.

 Sleep quality (PSQI): PSQI scores decreased significantly in the intervention group, indicating improved sleep (baseline: 9.1 ± 2.3; 3 months: 6.0 ± 1.7), whereas the control group showed only modest improvements (baseline: 9.0 ± 2.2; 3 months: 8.4 ± 2.0; *P* < .001, Cohen’s *d* = 1.23).

 Emotional distress (HADS): Total HADS scores at 3 months were significantly lower in the intervention group (8.3 ± 1.6 vs. 12.8 ± 2.3; mean difference: −4.5, 95% CI: −5.2 to −3.7; *P* < .001, Cohen’s *d* = 1.15), with consistent reductions observed in both anxiety and depression subscales.

These findings support the hypothesis that narrative therapy and resilience training enhanced emotional adaptation following ISR.

### Nutritional recovery

**Figure 3.**
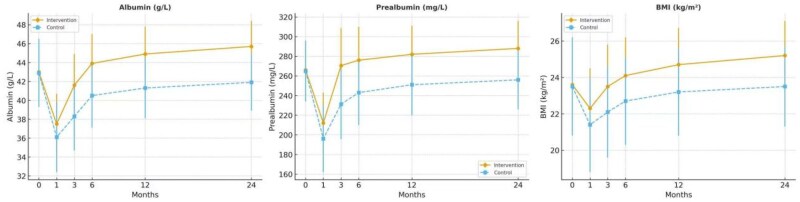
Postoperative nutritional outcomes. Notes: (A) Serum albumin levels declined after surgery in both groups but recovered more rapidly in the intervention group, returning to near-baseline by 3 months (mean difference: 3.3 g/L, 95% CI: 2.2-4.4; Cohen’s *d* = 0.96). (B) Serum prealbumin concentrations were consistently higher in the intervention group across all follow-up timepoints, reflecting enhanced short-term nutritional synthesis (Cohen’s *d* = 1.06 at 3 months). (C) Body mass index (BMI) recovered significantly faster in the intervention group compared with controls (mean difference at 3 months: 1.4 kg/m^2^, 95% CI: 0.6-2.2; *P* = .003; Cohen’s *d* = 0.56). All data are presented as mean ± standard deviation (SD), with between-group differences analyzed using independent-samples *t*-tests and Bonferroni correction for multiple comparisons. Error bars represent 95% confidence intervals (CIs) at each timepoint. Intervention fidelity and adherence were monitored throughout the study and are reported in the Results (*Psychosocial recovery: resilience, sleep, and emotional state* and *Nutritional recovery*). Detailed numerical results are provided in Supplementary Table S3.

Nutritional status, assessed via serum albumin, prealbumin, and BMI, deteriorated in both groups postoperatively but recovered significantly faster in the intervention group (Figure 3A-C; Supplementary Table S3):

1.  Albumin: At 3 months, levels returned to near-baseline in the intervention group (41.6 ± 3.1 g/L; 95% CI: 40.9-42.3) compared to persistently reduced levels in controls (38.3 ± 3.6 g/L; 95% CI: 37.4-39.2; mean difference: 3.3 g/L, *P* < .001, Cohen’s *d* = 0.96).
2.  Prealbumin: Levels were higher in the intervention group at all post-discharge timepoints, reaching 270.5 ± 38.1 mg/L at 3 months versus 231.2 ± 35.6 mg/L in controls (mean difference: 39.3 mg/L, 95% CI: 30.1-48.5; *P* < .001, Cohen’s *d* = 1.06).
3.  BMI: The intervention group demonstrated faster recovery in BMI (23.5 ± 2.3 vs. 22.1 ± 2.6 kg/m^2^ at 3 months; mean difference: 1.4, 95% CI: 0.7-2.1; *P* = .002, Cohen’s *d* = 0.56).

These results suggest that improved psychological status may facilitate metabolic and nutritional restoration following ISR.

### Postoperative recovery and complications

As detailed in Table 2, the intervention group experienced a shorter postoperative recovery period and fewer complications:

**Table 2.** Postoperative recovery outcomes.

Outcome	Intervention (*n* = 89)	Control (*n* = 89)	Statistic	*P*-value
**Hospital Stay (days)**	8.6 ± 1.9	10.1 ± 2.2	*t* = −4.92	<.001***
**Complication rate ≥ Grade II**	11 (12.4%)	21 (23.6%)	χ² = 4.44	.035*
**└ Postoperative ileus**	3 (3.4%)	6 (6.7%)	χ² = 1.12	.290
**└ Pulmonary infection**	2 (2.2%)	5 (5.6%)	χ² = 1.58	.208
**└ Wound complication**	2 (2.2%)	4 (4.5%)	χ² = 0.74	.391

Notes: Values are expressed as mean ± standard deviation (SD) for continuous variables and number (percentage) for categorical variables. Between-group comparisons for continuous variables were performed using unadjusted independent-samples *t*-tests, and categorical variables were analyzed using chi-square tests. No covariate adjustment was applied to the primary comparisons in this table.

* *P* < .05.

*** *P* < .001 indicate statistical significance. Missing data were addressed in sensitivity analyses using linear mixed-effects models (LMMs) under the intention-to-treat (ITT) principle.

1.  Length of hospital stay: Significantly reduced in the intervention group (8.6 ± 1.9 days vs. 10.1 ± 2.1 days; *P* < .001), reflecting enhanced recovery profiles.
2.  Major complications (Clavien–Dindo grade ≥ II): Occurred in 11 patients (12.4%) in the intervention group vs. 21 patients (23.6%) in the control group (*P* = .035). The most frequent events—postoperative ileus, wound infection, and urinary retention—were consistently lower in the intervention arm, although not all reached statistical significance individually.

These data indicate that improved emotional regulation and adherence to rehabilitation may translate into tangible surgical benefits.

### Systemic inflammation

Systemic inflammatory markers, measured on postoperative day 7, were significantly attenuated in the intervention group (Table 3):

**Table 3.** Postoperative inflammatory markers (mean ± SD).

Marker	Intervention (mean ± SD, 95% CI)	Control (mean ± SD, 95% CI)	Statistic	*P*-value
**CRP (mg/L)**	27.1 ± 6.4 (25.8-28.4)	34.9 ± 8.2 (33.0-36.8)	*t* = −6.56	<.001***
**IL-6 (pg/mL)**	20.9 ± 5.2 (19.6-22.2)	26.7 ± 6.3 (25.0-28.4)	*t* = −6.32	<.001***
**TNF-α (pg/mL)**	17.3 ± 4.3 (16.2-18.4)	22.0 ± 4.9 (20.7-23.3)	*t* = −5.91	<.001***

Notes: Values are presented as mean ± standard deviation (SD). Serum inflammatory markers (CRP, IL-6, TNF-α) were measured on postoperative day 7. Between-group comparisons were conducted using independent-samples *t*-tests, without covariate adjustment.

*** *P* < .001 indicate statistical significance. Sensitivity analyses using linear mixed-effects models (LMMs) were performed to account for repeated ­measurements over time.95% confidence intervals (CIs) for mean differences are reported in-text. All analyses followed the intention-to-treat (ITT) principle. Intervention fidelity and adherence were systematically monitored and are reported in the Results (*Psychosocial recovery: resilience, sleep, and emotional state* and *Nutritional recovery*).

1.  CRP: 27.1 ± 6.4 mg/L (95% CI: 25.8-28.4) vs. 34.9 ± 8.2 mg/L (95% CI: 33.0-36.8); *P* < .001, Cohen’s *d* = 1.08
2.  IL-6: 20.9 ± 5.2 pg/mL vs. 26.7 ± 6.3 pg/mL; *P* < .001, Cohen’s *d* = 1.00
3.  TNF-α: 17.3 ± 4.3 pg/mL vs. 22.0 ± 4.9 pg/mL; *P* < .001, Cohen’s *d* = 1.02

These findings suggest that the psychosocial intervention may modulate postoperative immune and stress responses, potentially mediating improved recovery and survival.

### Long-Term survival outcomes

Kaplan–Meier analysis (Supplementary Figure S1A and B) and multivariate Cox regression (Table 4) demonstrated significantly better long-term outcomes in the ­intervention group:

**Table 4.** Multivariate Cox regression analysis for DFS and OS.

Variable	HR (DFS)	95% CI (DFS)	*P*-value (DFS)	HR (OS)	95% CI (OS)	*P*-value (OS)
**Intervention (vs. control)**	0.52	0.30-0.91	.021*	0.46	0.23-0.94	.029*
**Age (per year)**	1.02	0.98-1.05	.321	1.01	0.98-1.04	.410
**Male sex**	1.12	0.71-1.76	.618	1.08	0.69-1.74	.730
**TNM stage (II/III vs. I)**	1.78	1.12-2.95	.017*	1.85	1.14-3.12	.015*
**Comorbidities (yes vs. no)**	1.24	0.81-1.91	.341	1.20	0.79-1.86	.367
**Postoperative complications (yes vs. no)**	1.47	1.05-2.06	.028*	1.52	1.07-2.14	.025*
**Baseline PSQI (per point)**	1.07	1.00-1.16	.043*	1.09	1.01-1.20	.032*
**Baseline albumin (g/L)**	0.92	0.86-0.98	.024*	0.89	0.83-0.97	.016*

Notes: Hazard ratios (HRs) and 95% confidence intervals (CIs) were calculated using multivariate Cox proportional hazards models for both Disease-Free Survival (DFS) and Overall Survival (OS). Models were adjusted for baseline covariates including age, sex, TNM stage, comorbidities, serum albumin, baseline psychological measures (PSQI, CD-RISC scores), and postoperative complication status.

* *P* < .05 indicates statistical significance. Model discrimination was evaluated using Harrell’s C-index (0.73 for DFS; 0.75 for OS), indicating good predictive accuracy. All analyses followed the intention-to-treat principle. Missing data were handled using multiple imputation (if applicable) or linear mixed-effects modeling as part of sensitivity analysis.

1.  2-year Disease-Free Survival (DFS): 89.2% vs. 75.3%; HR = 0.52, 95% CI: 0.30-0.91, *P* = .021
2.  2-year Overall Survival (OS): 93.1% vs. 81.6%; HR = 0.46, 95% CI: 0.23-0.94, *P* = .029

Multivariate models adjusted for age, sex, TNM stage, comorbidities, baseline albumin, and baseline psychosocial scores. Notably, postoperative complications were included as an independent covariate, and the analyses confirmed that complications were significantly associated with poorer DFS and OS. However, the intervention group continued to show a survival advantage even after adjusting for complications, indicating that the beneficial effects of the psychosocial program on long-term outcomes were independent of complication status.

### Subgroup analysis and summary of findings

Prespecified subgroup analyses (Supplementary Figure S2A and B) demonstrated that the intervention was associated with a consistent survival benefit across multiple clinically relevant strata, including age (per year), sex (male vs. female), TNM stage (II/III vs. I), comorbidity status (yes vs. no), baseline albumin, baseline CD-RISC (per 5 points), baseline HADS (per 5 points), baseline PSQI (per point), and protective diverting stoma (yes vs. no). The magnitude and direction of hazard ratios were generally concordant across these subgroups, and no significant interaction effects were detected (all *P* for interaction > .10).

Notably, patients with stage III disease, those with protective stomas, and individuals with higher baseline emotional distress (HADS ≥16) derived particularly clear improvements in both DFS and OS. These consistent findings across diverse clinical and psychosocial profiles reinforce the robustness and generalizability of the intervention. Importantly, because pelvic floor rehabilitation was uniformly incorporated into the standard ERAS pathway for both groups (Methods 2.3), the observed benefits can be attributed specifically to the added psychosocial components (narrative therapy and resilience training).

Together, these results underscore that the integrated intervention may enhance postoperative recovery and long-term oncologic outcomes irrespective of baseline demographic, clinical, or psychosocial characteristics.

### Summary of key findings

The integrated psychosocial intervention:

1.  Significantly improved psychological resilience, emotional well-being, and sleep quality;
2.  Accelerated nutritional and inflammatory recovery;
3.  Reduced postoperative complications and shortened hospitalization;
4.  Enhanced 2-year disease-free and overall survival after ISR.

## Discussion

This randomized controlled trial demonstrates that a structured psychosocial intervention—combining narrative therapy and resilience training—significantly improves psychological adaptation, accelerates functional and nutritional recovery, reduces systemic inflammation, and enhances long-term oncologic outcomes in patients undergoing intersphincteric resection (ISR) for low rectal cancer. These findings are particularly meaningful given the complex interplay between physical dysfunction and psychological distress that characterizes recovery after ISR, a technically sphincter-preserving but functionally disruptive procedure.

### Reframing recovery in ISR: beyond anatomical preservation

ISR aims to maintain sphincter integrity and avoid permanent stoma formation, yet it is frequently associated with debilitating bowel dysfunction, known collectively as low anterior resection syndrome (LARS).^20^ Symptoms such as urgency, fragmentation, and incontinence are not merely inconveniences but persistent sources of psychological burden, social withdrawal, and impaired quality of life. The routine creation of temporary stomas, though oncologically justified, further challenges patients’ body image and autonomy.^21^ Our study addresses this multidimensional recovery burden by integrating psychological support into postoperative care—a component often absent from conventional surgical protocols.

### Psychological resilience and recovery synergy

Resilience, defined as the capacity to maintain or regain mental health amid adversity, has emerged as a key determinant of cancer recovery. In our cohort, resilience training improved CD-RISC scores and contributed to sustained reductions in anxiety, depression, and sleep disturbances.^22^ These psychological improvements were maintained over 24 months, suggesting enduring emotional restructuring. The corresponding clinical benefits—shorter hospitalization, fewer complications, and improved nutritional trajectories—support a biopsychosocial model wherein emotional flexibility enhances physiologic recovery.^23^

### Narrative medicine: Restoring meaning amid bodily change

While resilience training strengthens internal coping mechanisms, narrative therapy enables patients to re-author their illness experience.^24^ For individuals recovering from ISR—often facing altered bodily function and disrupted identity—narrative engagement fosters emotional articulation and the reconstruction of meaning.^25^ The observed improvements in HADS and sleep quality may reflect resolution of internal conflict and existential distress, consistent with theories of expressive writing and cognitive reframing.

In addition, qualitative impressions from the psychologists who delivered the sessions highlighted several recurring themes: improved self-efficacy, reduced cancer-related stigma, greater acceptance of temporary stomas, and strengthened family communication. Although these insights were not systematically coded as formal qualitative data, they provide valuable experiential context that supports and enriches the quantitative improvements observed in resilience, emotional distress, and sleep outcomes.

### Psychoneuroimmunology and survival: bridging emotion and biology

A key mechanistic insight from our study is the significant attenuation of inflammatory markers (CRP, IL-6, TNF-α) in the intervention group.^26^ These findings support the psychoneuroimmunological model, wherein chronic psychological stress activates the HPA axis and sympathetic-adrenal-medullary system, promoting tumor-facilitating inflammation.^27^ By enhancing emotional regulation, our intervention likely interrupted this maladaptive cascade.

Importantly, although the structured psychosocial program formally commenced within two weeks postoperatively, preparatory counseling and orientation were initiated during the index hospital stay. This immediate engagement may have facilitated stronger adherence to ERAS elements (early mobilization, oral feeding, respiratory training) and reduced perioperative stress, thereby contributing to shorter length of stay, fewer complications, and lower systemic inflammatory response by postoperative day 7.

Moreover, our additional multivariate Cox regression analysis (Results 3.6, Table 4) incorporated postoperative complication status, along with age, sex, TNM stage, baseline albumin, and psychosocial scores. This analysis demonstrated that postoperative complications were independently associated with worse DFS and OS; however, the intervention group retained a significant survival advantage even after adjusting for complications. These findings suggest that the psychosocial intervention may exert direct benefits on long-term outcomes, beyond the reduction of perioperative complications.

This immune modulation may partly explain the observed improvement in long-term survival, consistent with emerging evidence linking psychosocial stability with micrometastatic control, tumor dormancy, and angiogenic balance.^28^^,^^29^

### Nutritional restoration as a psychophysiologic outcome

Nutritional status is a well-established determinant of surgical and oncologic outcomes. Malnutrition delays wound healing, impairs immunity, and increases complication risk. In our study, the intervention group demonstrated faster recovery of albumin, prealbumin, and BMI.^30^ This may reflect improved psychological readiness, better appetite regulation, and enhanced adherence to dietary recommendations. These results underscore the interconnectedness of emotional resilience and nutritional homeostasis along the gut–brain–immune axis.^31^

### Clinical integration: from adjunct to core strategy

Psychosocial interventions in oncology have historically been regarded primarily as adjunctive measures to enhance quality of life. Our findings extend this perspective by demonstrating that combined narrative therapy and resilience training were associated with superior 2-year DFS and OS, independent of TNM stage and baseline risk factors.^29^ These results challenge the traditional view of psychosocial care as peripheral, instead supporting its recognition as a core component of perioperative oncology with meaningful biologic and behavioral implications.

At the same time, we acknowledge that the conventional delivery of these interventions—particularly when provided as multiple, individualized sessions by trained professionals—can be resource-intensive and time-consuming. To address concerns about feasibility and scalability, the revised manuscript now highlights practical adaptations such as small-group delivery models, integration into existing ERAS pathways, and the use of telemedicine or digital storytelling platforms. These approaches could reduce resource demands, enhance efficiency, and facilitate broader implementation without compromising patient-centeredness.^32^^,^^33^

Moreover, the family education and support component—delivered through monthly seminars and supplementary materials—likely played a critical role in strengthening patient adherence to nutritional and rehabilitation protocols, fostering more effective family communication, and mitigating emotional distress. This interpretation is consistent with prior evidence demonstrating the positive influence of caregiver engagement on patient well-being and recovery in cancer care.^33–35^ Collectively, these insights suggest that embedding patient- and family-centered psychosocial strategies into perioperative practice may not only enhance emotional and functional recovery but also contribute to reduced complications, earlier discharge, and improved long-term oncologic outcomes.

### Implementation into surgical oncology pathways

The integration of psychosocial care into surgical oncology remains limited. Our findings suggest that structured psychological support can be feasibly embedded into perioperative protocols, particularly in high-risk groups such as ISR patients. More than 70% of our participants received temporary stomas, and all experienced LARS to varying degrees—yet meaningful improvements were observed across functional, psychological, and survival domains.^36^ These results echo international guidelines, including the NCCN Distress Management Guidelines and ASCO recommendations, which advocate for routine psychosocial assessment and intervention in cancer care.

### Limitations and future directions

Several limitations warrant consideration. First, the single-center design and moderate sample size may affect generalizability. Second, the relative contribution of narrative therapy versus resilience training remains unquantified.^37^ Third, biological markers such as cortisol, HRV, or lymphocyte profiles were not assessed, limiting mechanistic inference. Fourth, patients older than 75 years were excluded, primarily to minimize confounding from age-related cognitive impairment and frailty; however, we recognize that geriatric patients represent an important population that may derive substantial benefit from psychosocial interventions. Future studies should extend this research to elderly cohorts, incorporating tailored approaches to address the specific needs of this vulnerable group. Longer-term outcomes, including recurrence-free survival and functional independence, should also be evaluated.^38^

### Synthesis and broader implications

This study advances a paradigm in which postoperative recovery is viewed not simply as biological restitution, but as a psychosomatic reconstitution encompassing identity, emotion, function, and immune stability. For patients undergoing ISR—a surgery that preserves the sphincter but disrupts the self—narrative-based resilience training offers a reintegrative path.^39^ By recalibrating the mind–body axis, such interventions may not only alleviate distress but actively reshape the trajectory of cancer survivorship.^40^

## Conclusion

In this randomized controlled trial involving patients undergoing intersphincteric resection (ISR) for low rectal cancer, we demonstrated that an integrated psychosocial intervention—combining narrative therapy and resilience training—yielded clinically and biologically meaningful benefits across multiple domains of postoperative recovery. The intervention significantly improved psychological resilience, emotional stability, and sleep quality; accelerated nutritional reconstitution; reduced systemic inflammation; and translated into superior 2-year disease-free and overall survival. These findings validate the hypothesis that psychological adaptation is not only a determinant of quality of life, but also a modifiable driver of surgical and oncologic outcomes.

By targeting the emotional, cognitive, and existential disruptions associated with ISR—particularly in patients facing bowel dysfunction, temporary stomas, and identity challenges—the intervention addressed dimensions of recovery often overlooked in conventional postoperative protocols. The observed improvements in inflammatory and nutritional profiles provide mechanistic plausibility, linking psychosocial modulation to immune recovery and tumor control. Moreover, the intervention’s structure—non-invasive, low-cost, and scalable—supports its feasibility in routine clinical pathways.

Our results advocate for a paradigm shift in rectal cancer management: from a narrow focus on anatomical preservation toward an integrative approach that incorporates psychological rehabilitation as a core component of perioperative care. Future multicenter trials are warranted to validate these findings, explore long-term survival and functional trajectories, and refine stratified psychosocial interventions tailored to individual patient risk and needs.

Ultimately, this study reinforces that optimal recovery after ISR requires more than technical precision—it demands a holistic strategy that restores not only the bowel but also the person.

## Supplementary Material

oyaf308_Supplementary_Data

## Data Availability

All relevant data are included within this published article. Further details are available upon reasonable request.
