# Supplementary material for: Narrative therapy and resilience training improve recovery and survival after intersphincteric resection for low rectal cancer: a randomized trial
Source: Oncologist. 2025 Nov 4;30(11):oyaf308. doi: 10.1093/oncolo/oyaf308 (PMC12634408; doi:10.1093/oncolo/oyaf308)
Supplement: oyaf308_Supplementary_Data [file oyaf308_supplementary_data.zip › Supplementary Tables S1-3.docx]

Supplementary Table S1 - Overview of Intervention Components, Frequency, and Timepoints

| Component | Delivery Mode | Session Frequency | Session Duration | Timepoints | Total Sessions |
| --- | --- | --- | --- | --- | --- |
| Psychological Resilience Training (CBT-based) | Individual sessions | Once per week | 60 minutes | Weeks 1–8 post-surgery | 8 |
| Narrative Intervention | Small group (3–5 patients) | Biweekly | 90 minutes | Weeks 2, 4, 6, 8, 10, 12 | 6 |
| Family Education and Support | Group seminar + handouts | Monthly | 60 minutes | Months 1, 2, 3, 6, 12 | 5 |
| Follow-up Phone Counseling | Telephone | Every 2 months | 20–30 minutes | Months 3, 5, 7, 9, 11 | 5 |
| Standard Postoperative Care (Control group) | Routine clinical follow-up | N/A | N/A | As per clinical schedule | N/A |

Note: The psychological resilience training was delivered through structured, individual sessions based on cognitive behavioral therapy (CBT), focusing on adaptive coping, emotional regulation, and stress resilience. Narrative intervention consisted of facilitated small-group storytelling sessions aimed at reconstructing illness experiences and enhancing psychological growth. Family education involved monthly group seminars supplemented by printed handouts, covering emotional support, nutritional guidance, and colostomy adaptation. Follow-up telephone counseling was conducted every two months to reinforce intervention effects and provide ongoing psychosocial monitoring. The control group received standard postoperative care in accordance with institutional ERAS guidelines, without additional psychological or narrative components. [Reference: Gustafsson UO, Scott MJ, Hubner M, Nygren J, Demartines N, Francis N, et al. Guidelines for perioperative care in elective rectal/pelvic surgery: Enhanced Recovery After Surgery (ERAS®) Society recommendations. World J Surg. 2019;43(3):659–695.] Pelvic floor rehabilitation was included in the institutional ERAS pathway and was provided equally to both groups; therefore, it is not listed as a separate intervention component in the table.Intervention fidelity and adherence were monitored and are detailed in Results 3.2–3.3.

# Supplementary Table S2 - Postoperative Changes in Psychological Outcomes at Each Timepoint

| Outcome | Timepoint | Intervention Group (Mean ± SD) | Control Group (Mean ± SD) | p-value |
| --- | --- | --- | --- | --- |
| CD-RISC (Resilience Score) | Baseline | 59.8 ± 9.0 | 60.1 ± 8.8 | 0.780 |
| CD-RISC | 1 month | 67.3 ± 8.5 | 61.4 ± 9.1 | <0.001 |
| CD-RISC | 3 months | 74.3 ± 7.6 | 63.4 ± 9.5 | <0.001 |
| CD-RISC | 6 months | 75.8 ± 7.9 | 64.5 ± 9.2 | <0.001 |
| CD-RISC | 12 months | 77.1 ± 7.5 | 65.2 ± 9.0 | <0.001 |
| CD-RISC | 24 months | 77.9 ± 7.4 | 65.8 ± 8.9 | <0.001 |
| PSQI (Sleep Quality Score) | Baseline | 9.0 ± 2.4 | 9.1 ± 2.3 | 0.843 |
| PSQI | 1 month | 7.1 ± 2.0 | 8.6 ± 2.3 | <0.001 |
| PSQI | 3 months | 6.0 ± 1.7 | 8.4 ± 2.0 | <0.001 |
| PSQI | 6 months | 5.7 ± 1.6 | 8.1 ± 2.0 | <0.001 |
| PSQI | 12 months | 5.6 ± 1.5 | 7.9 ± 1.9 | <0.001 |
| PSQI | 24 months | 5.5 ± 1.4 | 7.7 ± 1.8 | <0.001 |
| HADS Total Score (Emotional Distress) | Baseline | 16.0 ± 4.1 | 16.3 ± 4.2 | 0.636 |
| HADS | 1 month | 10.2 ± 2.3 | 13.6 ± 2.4 | <0.001 |
| HADS | 3 months | 8.3 ± 1.6 | 12.8 ± 2.3 | <0.001 |
| HADS | 6 months | 8.0 ± 1.5 | 12.3 ± 2.2 | <0.001 |
| HADS | 12 months | 7.8 ± 1.4 | 11.9 ± 2.1 | <0.001 |
| HADS | 24 months | 7.6 ± 1.3 | 11.6 ± 2.0 | <0.001 |

Notes:Values are presented as mean ± standard deviation (SD). Between-group comparisons at each timepoint were conducted using independent-samples t-tests. Bonferroni correction was applied to adjust for multiple comparisons across repeated measures.All analyses followed the intention-to-treat (ITT) principle.

# Supplementary Table S3 - Postoperative Changes in Nutritional Outcomes at Each Timepoint

| Outcome | Timepoint | Intervention Group (Mean ± SD) | Control Group (Mean ± SD) | p-value |
| --- | --- | --- | --- | --- |
| Albumin (g/L) | Baseline | 43.0 ± 3.5 | 42.9 ± 3.6 | 0.79 |
|  | 1 month | 37.5 ± 3.2 | 36.1 ± 3.7 | 0.005 |
|  | 3 months | 41.6 ± 3.3 | 38.3 ± 3.6 | <0.001 |
|  | 6 months | 43.9 ± 3.1 | 40.5 ± 3.4 | <0.001 |
|  | 12 months | 44.9 ± 2.9 | 41.3 ± 3.2 | <0.001 |
|  | 24 months | 45.7 ± 2.7 | 41.9 ± 3.0 | <0.001 |
| Prealbumin (mg/L) | Baseline | 266 ± 30 | 265 ± 31 | 0.77 |
|  | 1 month | 212 ± 31 | 196 ± 34 | 0.002 |
|  | 3 months | 270.5 ± 38.1 | 231.2 ± 35.6 | <0.001 |
|  | 6 months | 276 ± 34 | 243 ± 33 | <0.001 |
|  | 12 months | 282 ± 29 | 251 ± 31 | <0.001 |
|  | 24 months | 288 ± 28 | 256 ± 30 | <0.001 |
| BMI (kg/m²) | Baseline | 23.6 ± 2.6 | 23.5 ± 2.7 | 0.68 |
|  | 1 month | 22.3 ± 2.2 | 21.4 ± 2.6 | 0.042 |
|  | 3 months | 23.5 ± 2.3 | 22.1 ± 2.5 | 0.003 |
|  | 6 months | 24.1 ± 2.1 | 22.7 ± 2.4 | <0.001 |
|  | 12 months | 24.7 ± 2.0 | 23.2 ± 2.4 | <0.001 |
|  | 24 months | 25.2 ± 1.9 | 23.5 ± 2.2 | <0.001 |

Notes:Values are presented as mean ± standard deviation (SD). Between-group comparisons at each timepoint were conducted using independent-samples t-tests, and Bonferroni correction was applied to account for multiple comparisons. All analyses followed the intention-to-treat principle.
