# Supplementary material for: Narrative therapy and resilience training improve recovery and survival after intersphincteric resection for low rectal cancer: a randomized trial
Source: Oncologist. 2025 Nov 4;30(11):oyaf308. doi: 10.1093/oncolo/oyaf308 (PMC12634408; doi:10.1093/oncolo/oyaf308)
Supplement: oyaf308_Supplementary_Data [file oyaf308_supplementary_data.zip › Supplementary Figure S1-2.docx]

Supplementary Figure S1 - Kaplan–Meier survival curves comparing the intervention and control groups.


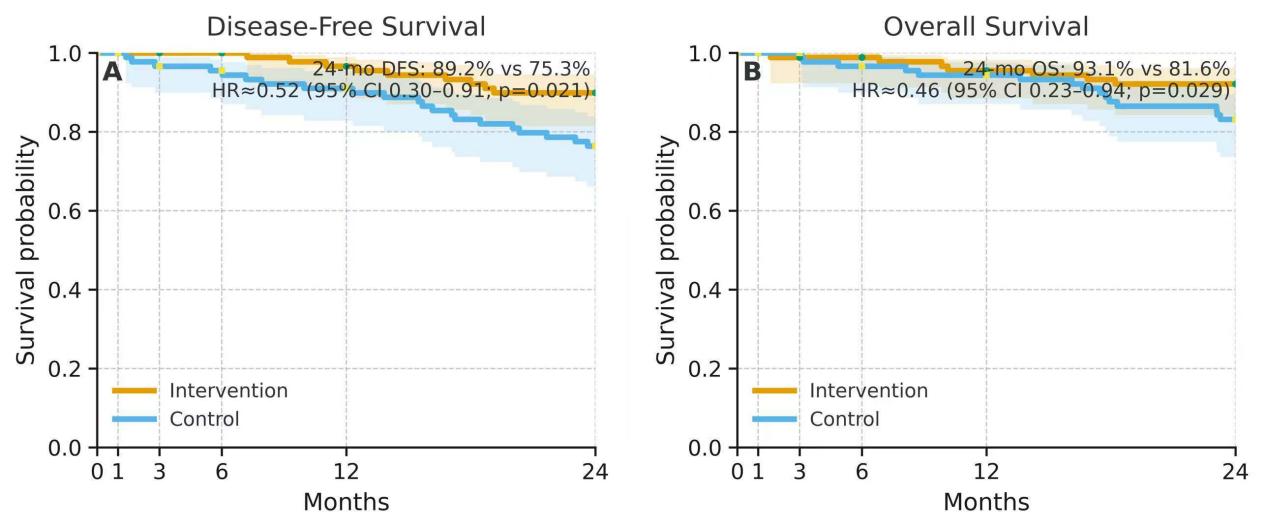


Notes: (A) Disease-free survival (DFS): The intervention group demonstrated significantly higher 24-month DFS (89.2% vs. 75.3%; hazard ratio [HR] = 0.52, 95% CI: 0.30–0.91; p = 0.021). (B) Overall survival (OS): The intervention group similarly exhibited improved 24-month OS (93.1% vs. 81.6%; HR = 0.46, 95% CI: 0.23–0.94; p = 0.029). Shaded bands represent 95% confidence intervals. Survival estimates were derived from multivariate Cox proportional hazards models adjusted for age, sex, TNM stage, baseline albumin, and baseline psychosocial scores (CD-RISC, HADS, PSQI). Both curves were extended to 24 months under the intention-to-treat (ITT) principle. The proportional hazards assumption was verified and not violated.

Supplementary Figure S2 - Subgroup analyses of intervention effects on survival after intersphincteric resection (ISR).


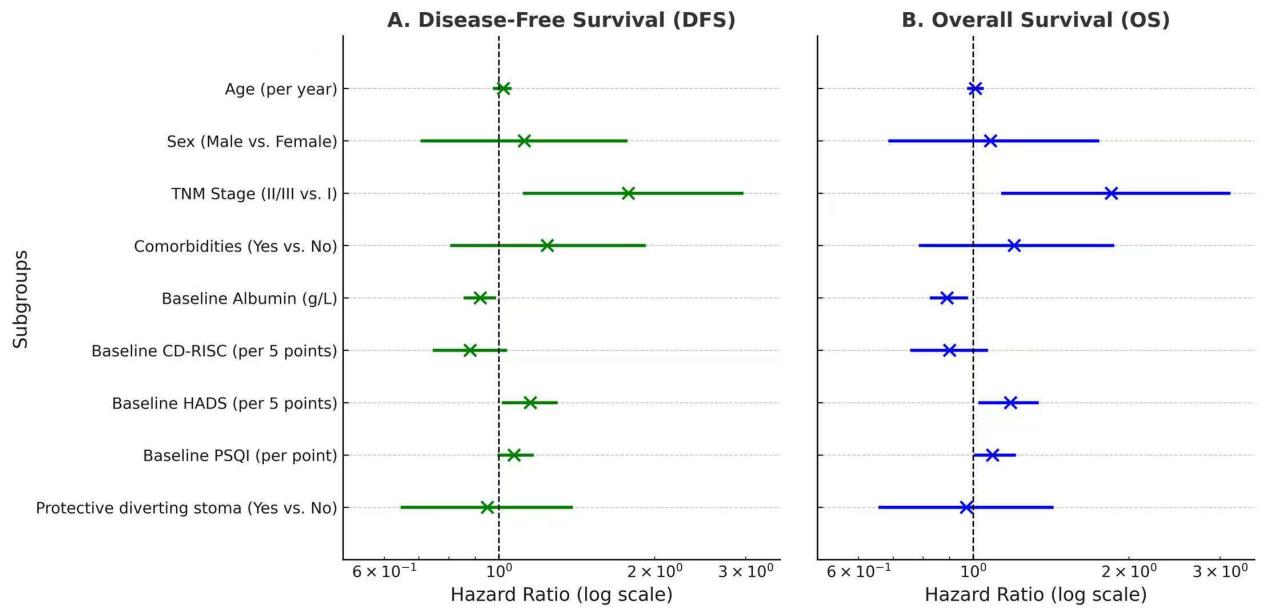


Notes: (A) Forest plot of hazard ratios (HRs) with 95% confidence intervals (CIs) for 2-year disease-free survival (DFS). (B) Forest plot of hazard ratios (HRs) with 95% CIs for 2-year overall survival (OS).The vertical dashed line indicates HR = 1 (no effect). Points represent HR estimates, and horizontal lines indicate 95% CIs. Values to the left of the line favor the intervention group, while those to the right favor the control group. Subgroups are shown on the Y-axis: Age (per year), Sex (Male vs. Female), TNM Stage (II/III vs. I), Comorbidities (Yes vs. No), Baseline Albumin (g/L), Baseline CD-RISC (per 5 points), Baseline HADS (per 5 points), Baseline PSQI (per point), and Protective diverting stoma (Yes vs. No).Hazard ratios were estimated using multivariate Cox proportional hazards models adjusted for baseline covariates, including age, sex, TNM stage, comorbidities, serum albumin, and baseline psychological measures (PSQI, CD-RISC, HADS). *P < 0.05 indicates statistical significance. Model performance was evaluated using Harrell’s C-index (0.73 for DFS; 0.75 for OS).
